# Supplementary material for: Helicase promotes replication re-initiation from an RNA transcript
Source: Nat Commun. 2018 Jun 13;9:2306. doi: 10.1038/s41467-018-04702-x (PMC5997990; doi:10.1038/s41467-018-04702-x)
Supplement: Supplementary file 1 — Supplementary Information [file 41467_2018_4702_MOESM1_ESM.pdf]

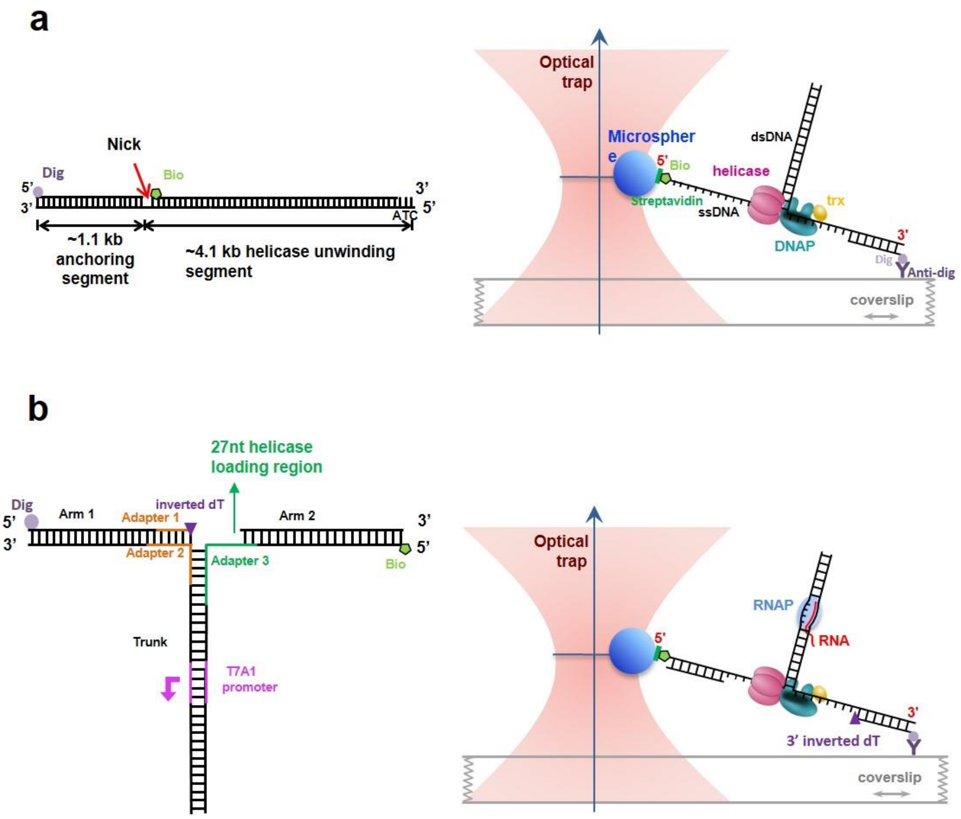

Supplementary Figure 1. DNA template designs and experimental configurations.

a, DNA template design and experimental configuration for helicase unwinding and dsDNA unzipping assay. A 5.2 kb DNA was constructed as described in Methods. One strand of the dsDNA to be unzipped/unwound was attached to a trap microsphere via a biotin/streptavidin connection. The other strand was anchored to be a microscope coverslip surface via a dig/anti-dig connection. The trapped microsphere was held in a feedback-enhanced optical trap so that its position relative to the trap center and the trapping force could be measured. The two strands were then held under a constant force. Helicase unwinding and slippage in the presence and absence of a non-replicating DNAP was monitored as an increase and decrease in the DNA length respectively[1, 2].

b, DNA template design and experimental configuration for helicase-DNAP coupled replication re-initiation assay. The DNA construct was made from three DNA segments: two arms and a trunk, linked through three short adapters (see Methods). A 3' inverted dT was incorporated at the adapter 1 (IDT) from which DNAP could not synthesize. The purple arrow indicates the transcriptional direction of RNAP from the promoter. Similarly, two arms of the DNA construct were attached to a trapped

microsphere and a microscope coverslip respectively. Helicase unwinding and helicase-DNAP coupled DNA synthesis were monitored as a change in the DNA length.

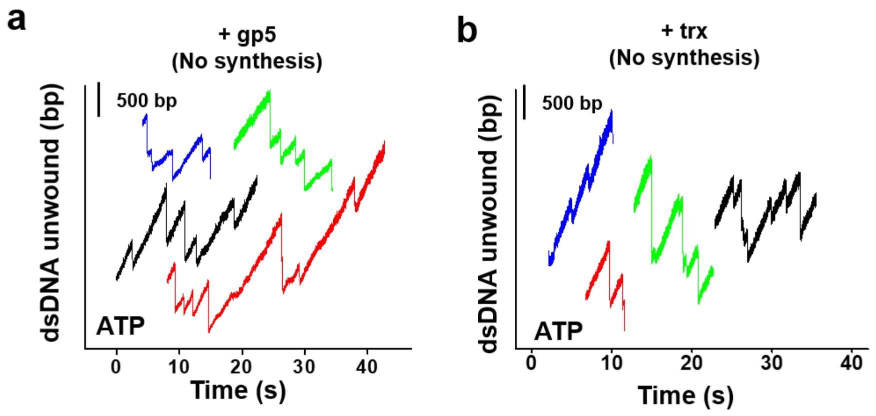

Supplementary Figure 2. Helicase unwinding in the presence of T7 gp5 or Trx.

Representative traces showing the unwound base pairs of dsDNA by T7 helicase versus time in the presence of either T7 gp5 **(a)** or trx **(b)** under 2 mM ATP and 8 pN. In both cases, T7 helicase exhibited slippage behavior and its processivities are comparable to that in the absence of gp5 and trx (Fig. 1d). These results, in addition to those of Figure 2b, demonstrate both gp5 and trx are required to prevent T7 helicase from slippage.

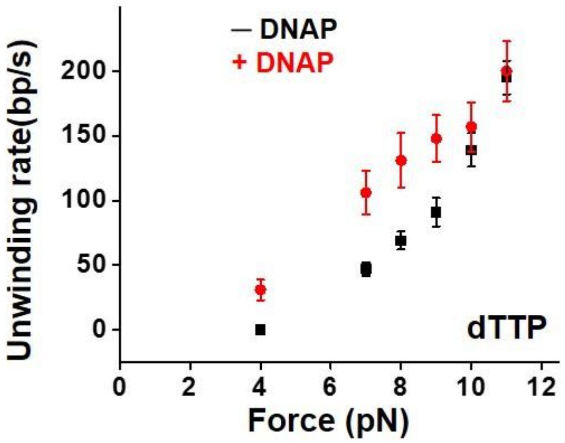

Supplementary Figure 3. Helicase unwinding rates as a function of force in the absence and presence of non-replicating DNAP with dTTP.

The experiments were conducted with 2 mM dTTP. From 4 pN to 10 pN, T7 helicase increased its unwinding rates with the presence of a non-replicating DNAP. Notably, T7 helicase could hardly unwind under 4 pN under our experimental condition, whereas it showed relatively strong unwinding activity when the DNAP was present. At 12 pN, the unwinding rates were comparable with or without the DNAP and were also close to helicase's ssDNA translocation rate[2], suggesting fork was no longer a substantial barrier for helicase translocation.

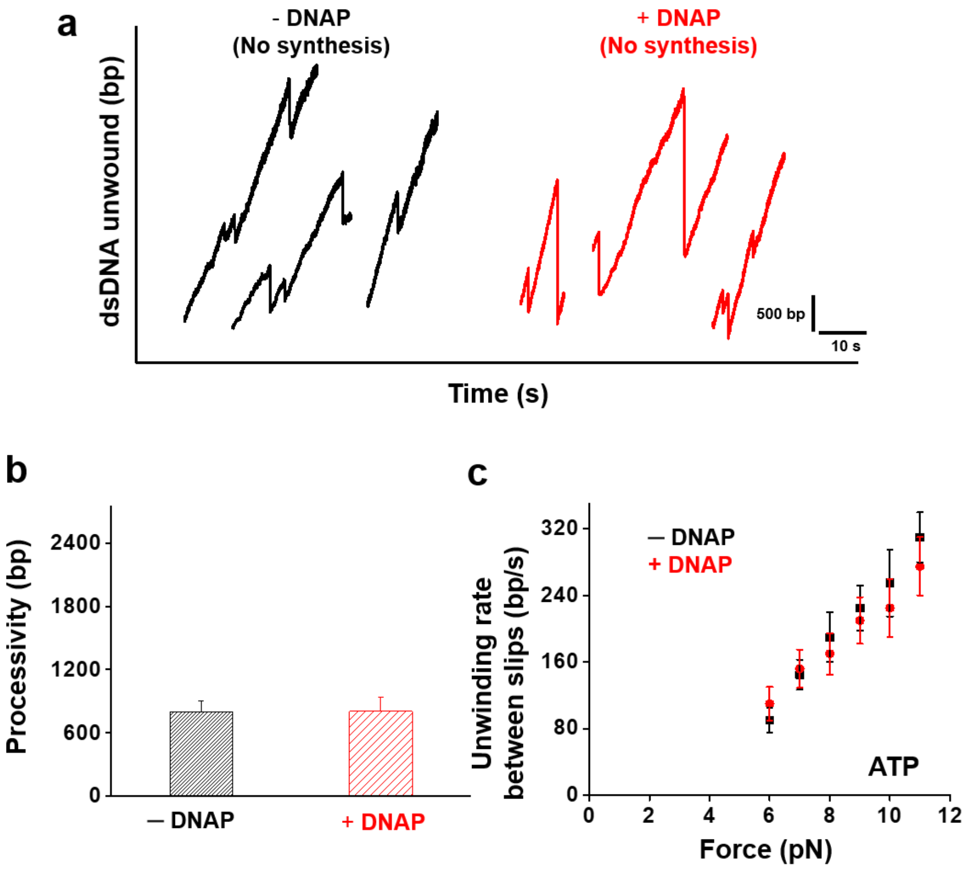

Supplementary Figure 4. Non-replicating DNAP does not regulate ΔCt helicase mutant unwinding.

a, Representative traces showing the number of unwound base pairs by ΔCt helicase versus time in the absence and presence of the non-replicating DNAP. Experiments were conducted with 3 mM ATP and 0.2 mM dTTP under 7 pN.

b, Measured processivity of ΔCt helicase in the absence and presence of the non-replicating DNAP with 3 mM ATP and 0.2 mM dTTP and 7 pN.

c, ΔCt unwinding rate between slips as a function of force in the absence and presence of the non-replicating DNAP with 3 mM ATP and 0.2 mM dTTP.

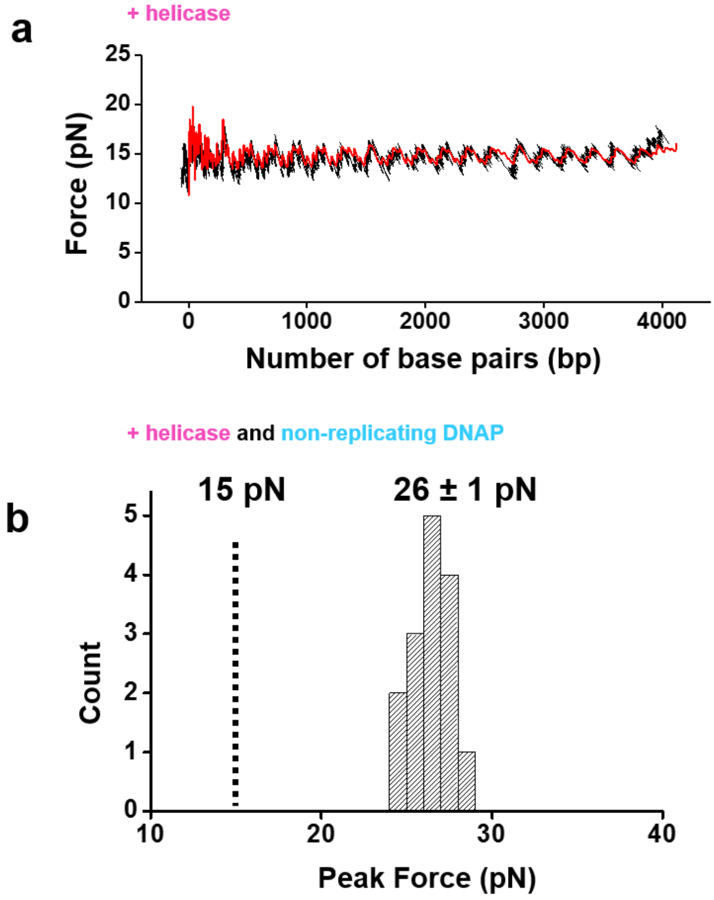

Supplementary Figure 5. Peak force distribution in the presence of non-replicating DNAP and helicase

a. Representative trace showing the force versus number of base pairs mechanically unzipped in the presence of the T7 helicase only. The experiments were conducted with 30 nM T7 helicase. The entire dsDNA template was mechanically unzipped at 600 bp/s. The red trace corresponds to the theoretical unzipping force of naked DNA of the same sequence[2-4]. The measured unzipping force in the presence of the helicase (in black) is in good agreement with the theoretical force, indicating that T7 helicase exhibits minimal interactions with dsDNA and the force rise in the presence of both non-replicating DNAP and helicase was not due to the helicase only. This observation is consistent with that of Figure 2a.

b. Peak force distribution. The peak force observed in the unzipping trace in the presence of both DNAP and helicase as outlined in Figure 2b is 26 ± 1 pN on average, substantially above the naked DNA force baseline with or without helicase (15 pN, back dashed line).

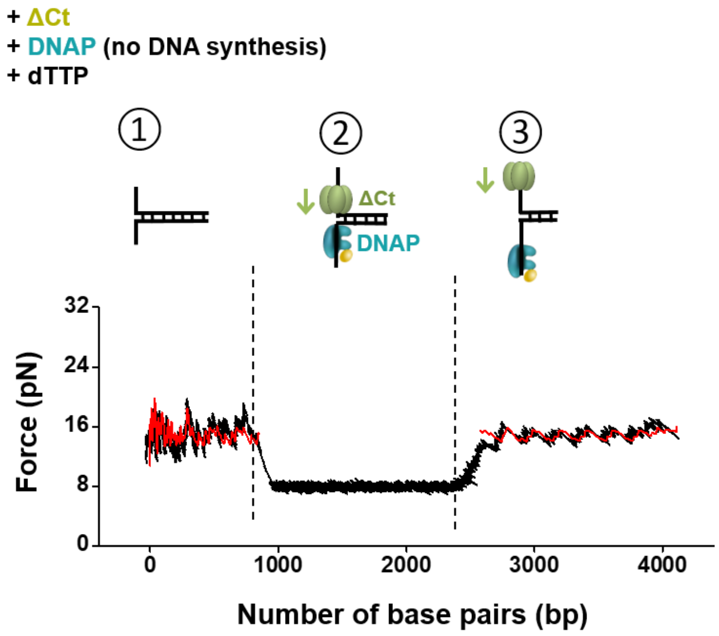

Supplementary Figure 6. Unzipping signature of dsDNA with ΔCt helicase in the presence of non-replicating DNAP.

Representative trace showing the force versus number of base pairs unzipped/unwound in the presence of the non-replicating DNAP and Δ Ct helicase. The red curve corresponds to unzipping naked DNA. The experimental procedure was described in the main text. Under this condition and during step 3, no force rise significantly above the naked DNA baseline was observed, suggesting that the force rise events observed with wild type T7 helicase and the DNAP was a results of direct interactions between them at the fork.

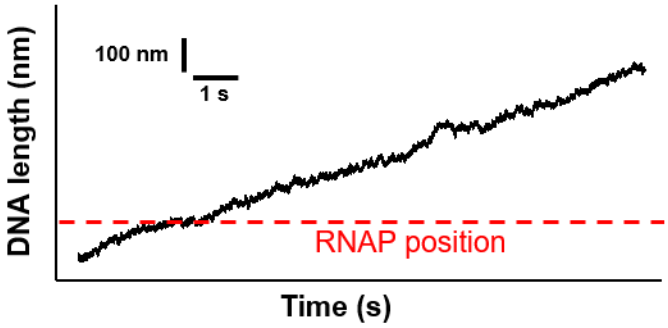

Supplementary Fig. 7: T7 helicase alone unwinds and displaces RNAP.

A representative trace of helicase unwinding through stalled TEC in the absence of non-replicating DNAP. To test whether T7 helicase alone is able to displace RNAP, we performed helicase unwinding assay at 0.5 mM dNTP (each) under 5 pN of force on a DNA template containing a co-directional TEC stalled at +20 nt from its promoter. The dotted line indicates the expected stalled TEC position. We found that, in the absence of non-replicating DNAP, T7 helicase alone is able to unwind and displace RNAP.

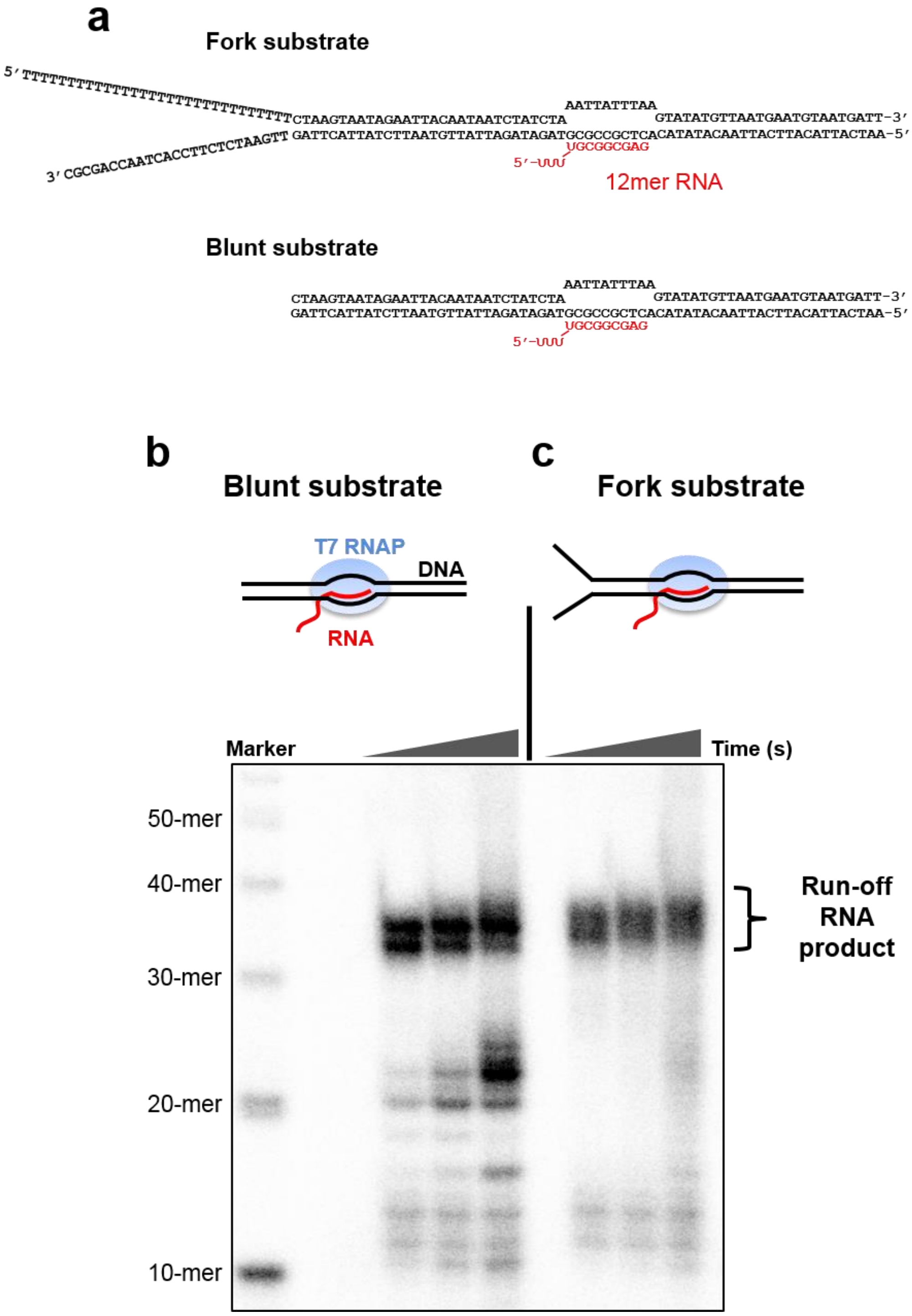

Supplementary Fig. 8: T7 TEC can make run-off RNA products.

The DNA and RNA sequences used for forming the fork and blunt substrates are shown in Supplementary Fig. **8a**. To ensure that T7 TEC is properly formed on the blunt **(b)** and fork **(c)** substrates used in the ensemble assays, control experiments were performed at 18°C with T7 RNAP (1100 nM), fork or blunt substrate (200 nM)

containing RNA primer (12-mer RNA) in reaction buffer (50 mM Tris-Cl pH 7.5, 40 mM NaCl, 10% glycerol, 2 mM DTT). Reactions were initiated by adding 200 μM rNTPs and 10 mM $MgCl_2$, with the rNTP mixture spiked with [α-^{32}P]-ATP. . For each experiment, the reactions were quenched at set time points (0, 60, 180 and 600 s) with 0.15 M final concentration of EDTA. Samples were added to formamide and bromophenol blue dye and heated at 95°C for 5 minutes before loading on 12% polyacrylamide/6 M urea sequencing gels. Gels were exposed to phosphor screens and scanned on Typhoon FLA 9500 scanner (GE Healthcare). The gel image shows the kinetics of the RNA primer extension on both substrates. The expected 38-mer run-off RNA products for both substrates indicate that TEC was formed properly.

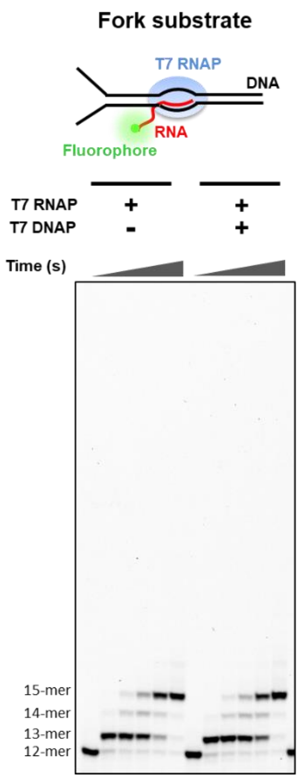

Supplementary Figure 9. Addition of dNTPs to the fluorescein labeled primer by T7 RNAP in absence and presence of DNAP.

Reactions were performed on TEC formed on fork substrate. TEC substrate with fluorescein labeled primer (200 nM) was incubated with T7 RNAP (1100 nM) and exo- T7 DNAP (220 nM). Reactions were initiated by addition of dNTPs (200 µM each) and $MgCl_2$ (10 mM). For each experiment, reactions were quenched at preset time points (0, 5, 60, 180, 600 and 3600 s) with addition of EDTA (0.2 M). Samples were mixed with formamide and bromophenol blue dye and heated at 95ºC for five minutes. Denatured samples were resolved on a 7 M urea/24% acrylamide sequencing gel. Gel was scanned for fluorescein signal on Typhoon FLA 9500 scanner. The result shows that T7 RNAP incorporates dNTPs and extends the fluorescein labeled 12-mer primer to 15-mer.

Supplementary References

1. Sun, B. et al. T7 replisome directly overcomes DNA damage. *Nat Commun* **6**, 10260 (2015).
2. Johnson, D.S., Bai, L., Smith, B.Y., Patel, S.S. & Wang, M.D. Single-molecule studies reveal dynamics of DNA unwinding by the ring-shaped T7 helicase. *Cell* **129**, 1299-1309 (2007).
3. Essevaz-Roulet, B., Bockelmann, U. & Heslot, F. Mechanical separation of the complementary strands of DNA. *Proc Natl Acad Sci U S A* **94**, 11935-11940 (1997).
4. Bockelmann, U., Thomen, P., Essevaz-Roulet, B., Viasnoff, V. & Heslot, F. Unzipping DNA with optical tweezers: high sequence sensitivity and force flips. *Biophys J* **82**, 1537-1553 (2002).
